# Chronic Social Defeat Stress Up-Regulates Spexin in the Brain of Nile Tilapia (Oreochromis niloticus)

**DOI:** 10.1038/s41598-020-64639-4

**Published:** 2020-05-06

**Authors:** Chor Hong Lim, Tomoko Soga, Berta Levavi-Sivan, Ishwar S. Parhar

**Affiliations:** 1 Brain Research Institute, Jeffrey Cheah School of Medicine and Health Sciences, Monash University Malaysia, 47500 Selangor, Malaysia; 2 Department of Animal Sciences, The Robert H Smith Faculty of Agriculture, Food, and Environment, Hebrew University of Jerusalem, Rehovot, 76100 Israel

**Keywords:** Chemical biology, Neuroscience

## Abstract

Spexin (SPX), a neuropeptide evolutionarily conserved from fish to mammals, is widely distributed in the brain and peripheral tissues and associated with various physiological functions. Recently SPX has been suggested to be involved in neurological mechanism of stress. The current study investigates the involvement of SPX in chronic social defeat stress, using male teleost, the Nile tilapia (Oreochromis niloticus) as an animal model due to its distinct social hierarchy of dominant and subordinate relationship. The tilapia genome has SPX1a and SPX1b but has no SPX2. In the Nile tilapia, we localized SPX1a and SPX1b in the brain using *in-situ* hybridization. Next, using qPCR we examined gene expression of SPX1a and SPX1b in chronically stress (socially defeated) fish. SPX1a expressing cells were localized in the semicircular torus of the midbrain region and SPX1b expressing cells in the telencephalon. Chronically stress fish showed elevated plasma cortisol levels; with an upregulation of SPX1a and SPX1b gene expression in the brain compared to non-stress (control) fish. Since social defeat is a source of stress, the upregulated SPX mRNA levels during social defeat suggests SPX as a potentially inhibitory neuropeptide capable of causing detrimental changes in behaviour and physiology.

## Introduction

Spexin (SPX) is a neuropeptide that consists of 14 amino acids and is conserved across vertebrates^1^. Using synteny analysis and data mining, a novel spexin (SPX2) has been reported in the genome of non-mammalian vertebrates, except in cichlids and mammals^2,3^. In mammals, SPX is widely expressed in various brain regions and peripheral tissues^4^. Parallel to its wide distribution across different brain regions, SPX has been associated with multiple physiological functions such as reproduction, nociception, cardiovascular, metabolic homeostasis, feeding behaviors and obesity^4^. In cichlids the sequence of SPX mature peptide is three amino acids different from the original SPX1^3^. Hence, the original SPX1 is termed SPX1a and the newly found gene as SPX1b^3^. (The cloned sequence of SPX1a and SPX1b is indicated in Supplementary Data 1).

The coevolution of SPX and galanin (GAL) peptide family and the finding that SPX can activate galanin receptor 2 (GALR2) and galanin receptor 3(GALR3) has drawn attention to the possible interactive role between these two neuropeptides^2^. GAL and GALRs have been reported to be associated with depression-like behavior^5^. Given the proactive role played by GAL and its receptor in depression/anxiety; the role of spexin in depression can be speculated. In fact, rats treated with escitalopram, an antidepressant, show changes in SPX gene expression in the brain, with SPX being upregulated in the hippocampus and striatum but downregulated in the hypothalamus^6^. Furthermore, in the placental samples of mothers with antenatal depression, SPX gene expression is down-regulated and the expression level can be normalized by antidepressant treatment^7^. Recently, SPX has also been shown in the medial habenula of mice and dorsal habenula of the zebrafish^8,9^, an evolutionary conserved brain area involved in the regulation of depression and anxiety^10,11^. Stressful life events are key contributors to the onset of major depression^12^. The hypothetical role of SPX in the mechanism of anxiety and depression is also speculated from its localization in brain regions that are involved in the neurobiology of stress. SPX is localized in the Barrington’s nucleus, hypothalamic paraventricular nuclei and locus coeruleus in the rat^13^. Previous studies have linked these three brain regions to play an inter-connected role in the regulation of stress responses^14,15^, and the presence of SPX in these brain regions have led to the hypothesis that SPX could, acting via GARL2/3, contribute to the dysregulation of the HPA axis during stressful stimuli.

Social defeat stress refers to stress induced by conflict between members of the same species. The social defeat stress paradigm is commonly induced by exposing a male subject into the resident of a bigger and more aggressive dominant male^16^. Social defeat stress has been shown to induce behavioral and psychological changes in human subjects^16^. Experience of social defeat stress can also lead to suicidal behavior and mood disorder in victims of bullying^17^. The social defeat stress paradigm draws its strength on resembling the conflicts in human settings which are social in characteristic. The biological changes observed after social defeat stress are dependent upon the frequency of exposure to stress^18,19^. Furthermore, changes in neuronal mechanism and structural changes have been shown in the brain of socially defeated animals. In rodents, neurogenesis, as well as the volume of the hippocampus and the medial prefrontal cortex, are suppressed after exposure to defeat stress^20,21^. Similarly, in teleosts, exposure to social stress can impair neurogenesis and cell proliferation in the ventral part of the telencephalon in subordinate male zebrafish^22^. Dysregulation of the hypothalamic–pituitary–adrenal (HPA) axis has been identified as a risk factor that contributes to the onset of depression^23^. Rodents exposed to social defeat stress demonstrate signs of HPA axis dysregulation, such as upregulation of glucocorticoid levels, ACTH and corticosterone levels^24^. Coherent observation is also reported in Nile tilapia and zebrafish where social defeat stress increases plasma cortisol levels^22,25^.

We hypothesize that SPX may be involved in the social defeat stress. To test this hypothesis, we used cichlid, Nile tilapia (Oreochromis niloticus) a fish species, which displays social hierarchy, with a dominant and a subordinate male that change to darker skin coloration with increasing stress^25^. In the Nile tilapia, we localized SPX1a and SPX1b in the brain using *in-situ* hybridization (ISH). Next, using real-time PCR we examined gene expression of SPX1a and SPX1b in chronically stress (socially defeated) (two hours stress daily over 5 days).

## Results

### Plasma cortisol level

In chronic social defeat stress experiment, the plasma cortisol levels of defeated (stress) fish was significantly higher than the control (non-stress) group (139.91 ng/ml ± 16.91 vs 58.38 ng/ml ± 2.97) (fold change: 2.40; *p* = 0.014).

### Distribution of SPX1a & SPX1b gene expression in the brain of nile tilapia

Using qPCR (Fig. 2A), SPX1a mRNA expression was found to be highest in Area 2 (optic tectum, hypothalamus and midbrain), followed by Area 3, and lowest in Area 1 (Fig. 2B). There was significant difference between Area 2 &3 and Area 1 (*p* < 0.001). In contrast, the highest expression of SPX1b was found in the Area 1 (telencephalon and preoptic area), followed by Area 3 and Area 2 and there was significant difference between Area 1 and Area 2 (*p* = 0.015) (Fig. 2C). The copy number of SPX1b (12048 copy/ 1000 ng RNA) was lower compared to SPX1a (480570 copy/1000 ng RNA).

**Figure 1:**
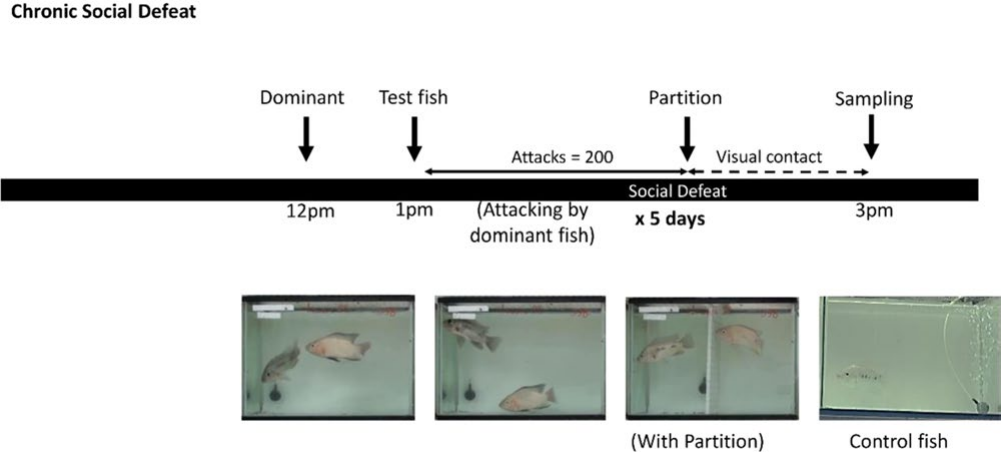
Social Defeat experiment paradigm. For chronic social defeat, the test fish was exposed to social defeat stress for 2 hours daily for 5 consecutive days. Sampling for defeated and control fish was done at the same timing at the 5^th^ day. Control group was exposed to the same protocol with the absence of the dominant fish.

**Figure 2:**
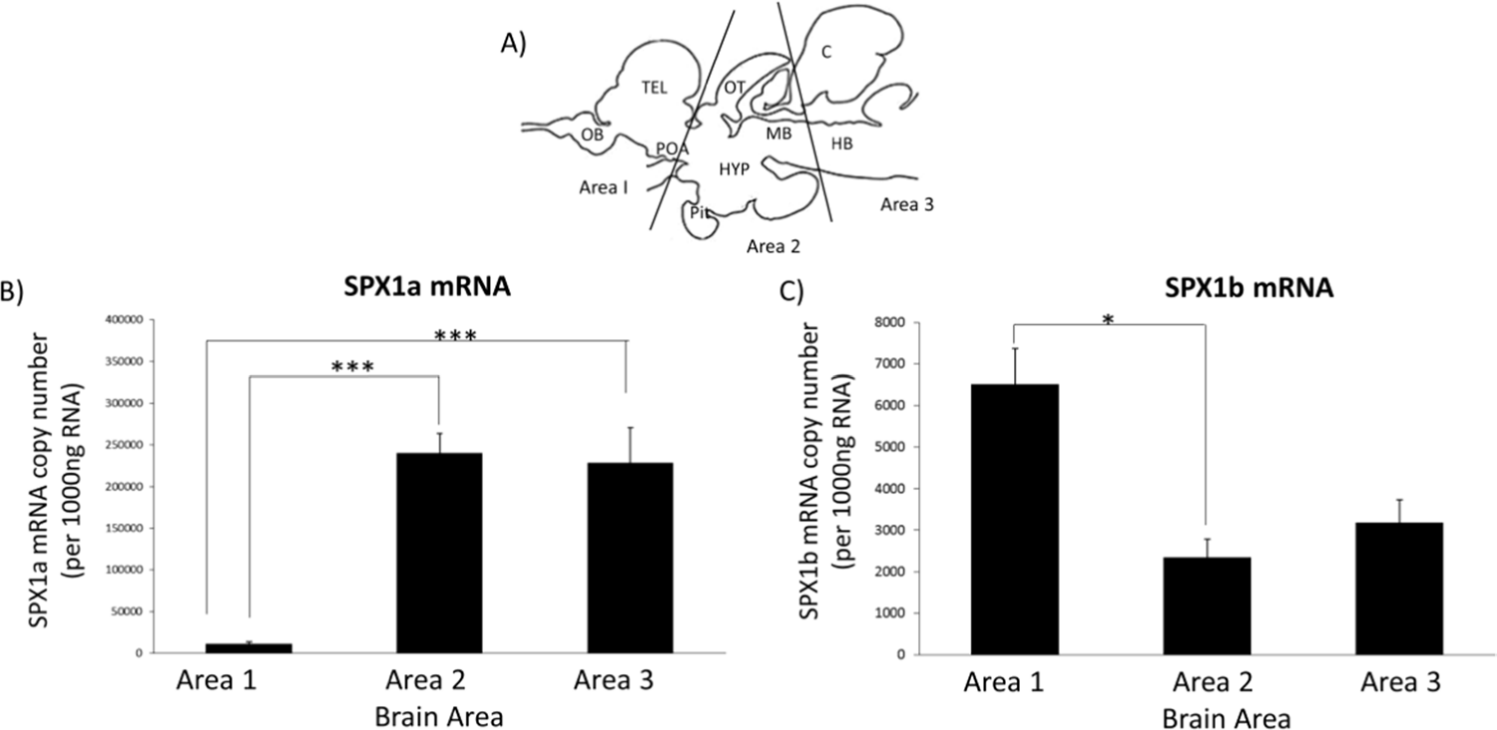
Distribution of SPX1a and SPX1b in the brain of Nile tilapia. (**A**) Dissection of the brain into 3 areas. OB, olfactory bulb; POA, preoptic area; TEL, telencephalon; OT, optic tectum; HYP, hypothalamus; PIT, pituitary; MB, midbrain; C, cerebellum; HB, hindbrain. (**B**) Distribution of SPX1a mRNA is highest in area 2. (**C**) Distribution of SPX1b is highest in area 1. (*p < 0.05, ***p < 0.001).

### Localization of SPX1a & SPX1b in the brain of Nile tilapia

**Figure 3:**
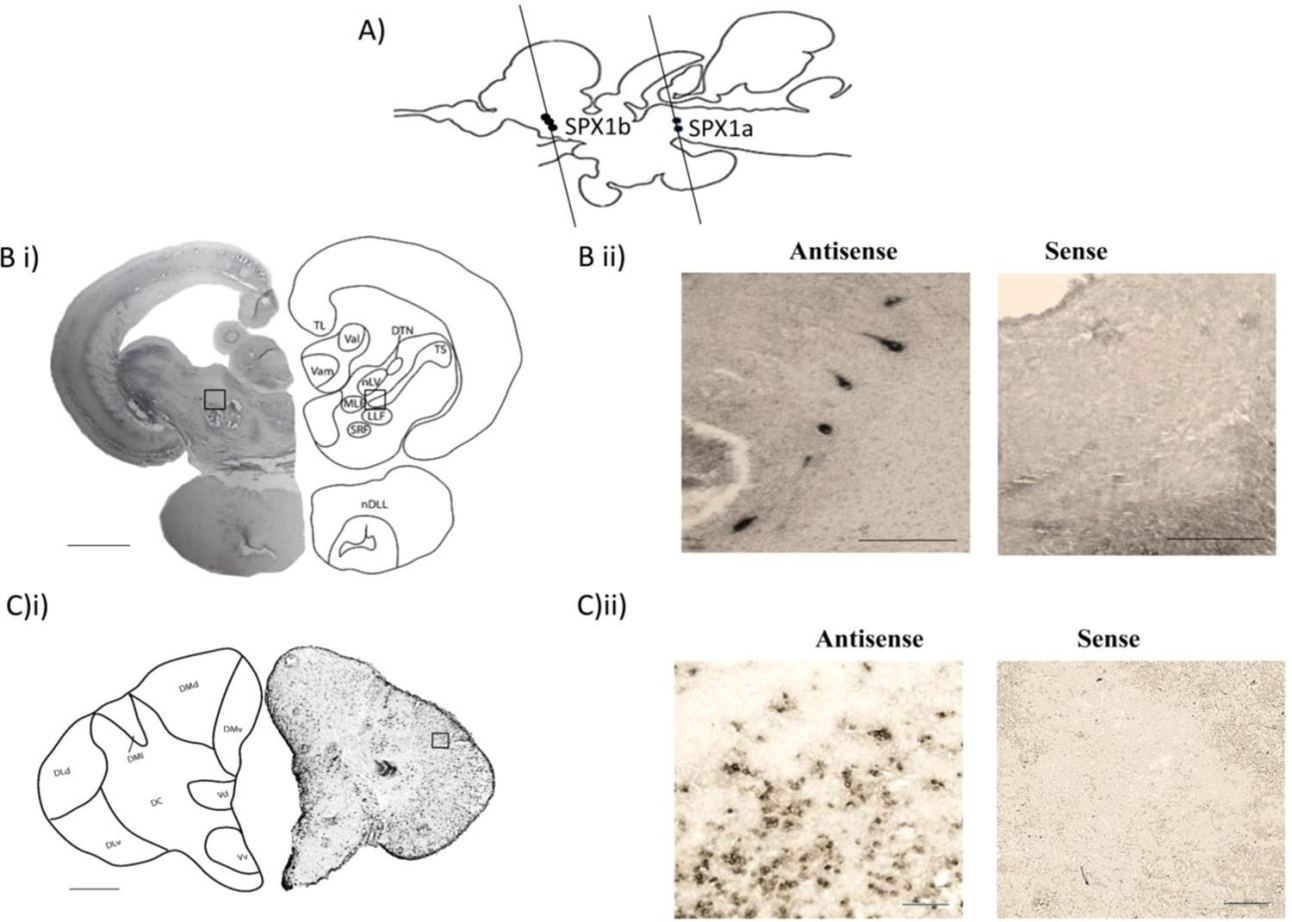
Localization of SPX1a and SPX1b expression in the Nile tilapia brain. (**A**) Sagittal section showing the signal of SPX1a and SPX1b with the relative position of the coronal section. (**B**) i) Coronal section showing the signal of SPX1a. SPX1a was localized in the ventromedial nucleus of semicircular torus (TS). (Scale bars = 500 μm). (**B**) ii) Photomicrographs of the signal with 20x magnification. (Scale bars = 100 μm) .(**C**) i) Coronal section showing the signal of SPX1b. SPX1b was localized in the telencephalon, with heavy staining observed in the lateral part of the dorsal telencephalon. (Scale bars = 500 μm). (**C**) ii) Photomicrographs of the signal with 40x magnification. (Scale bars = 50 μm) TL, longitudinal torus, Val, Lateral valvula cerebelli, Vam, Medial valvula cerebelli, DTN, Dorsal tegmental nucleus, nLV, Lateral valvular nucleus, mLF, medial longitudinal fascicle, SRF, superior reticular formation, LLF Lateral longitudinal fascicle, TS, semicircular torus, nDLL, diffuse nucleus of the lateral lobe. Dmd, medio-dorsal area of dorsal telencephalon; Dmv, medio-ventral area of dorsal telencephalon; DM1, Subdivision 1 of the medial zone of the dorsal telencephalon; DC, central zone; DLd, dorsal subdivision of lateral part of dorsal telencephalon; DLV, ventral subdivision of lateral part of dorsal telencephalon; Vd, dorsal part of the ventral telencephalon; Vv, ventral nucleus of the ventral telencephalon.

ISH showed SPX1a positive cells localized in the midbrain and SPX1b positive cells localized in the telencephalon (Fig. 3A). The staining with sense RNA probe did not exhibit any hybridization signal, while staining with anti-sense RNA probe show clear signal, indicating the specificity of the RNA probe.

Coronal sections revealed that SPX1a expressing cells were specifically localized in the semicircular torus (TS), and close examination revealed that there were located in the ventromedial nucleus of the TS (Fig. 3B). Within the dorsal telencephalon, all subdivisions including the dorsal, medial and the lateral zone (medio-dorsal area of dorsal telencephalon (DMd); medio-ventral area of dorsal telencephalon (DMv); Subdivision 1 of the medial zone of the dorsal telencephalon (DM1); dorsal subdivision of lateral part of dorsal telencephalon (DLd); ventral subdivision of lateral part of dorsal telencephalon (DLv) had SPX1b positive cells, while lateral subdivision of the dorsal telencephalon (DLd, DLv) had cells with higher staining intensity compared to other subdivisions. Cells in the central zone of the dorsal telencephalon (DC) showed weak and scattered staining for SPX1b. Moderate staining was also observed in the subdivisions of the ventral telencephalon including the dorsal part of the ventral telencephalon (Vd); ventral nucleus of the ventral telencephalon (Vv) (Fig. 3C).

### Gene expression of SPX1a & SPX1b under chronic social stress

**Figure 4:**
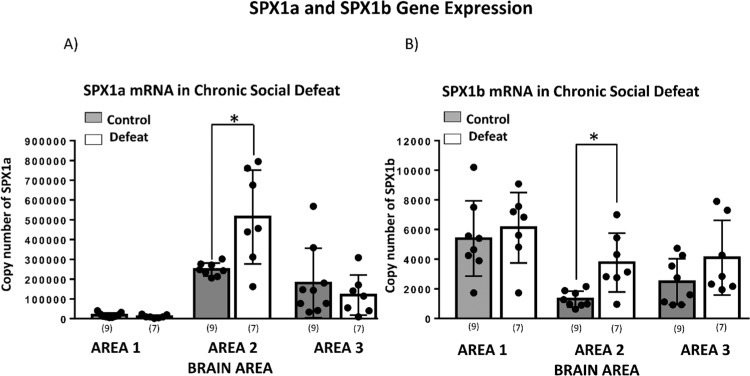
SPX1a and SPX1b gene expression in chronic social defeat. (**A**) SPX1a was upregulated in area 2 after chronic social defeat. (**B**) SPX1b was also upregulated in area 2 after chronic social defeat. All data are expressed as the mean ± SEM per group, and independent sample T-test was carried out using SPSS. Statistical significance was defined as *P* value less than 0.05. (*p < 0.05).

Gene expression analysis of both SPXs in the chronic social defeat stress revealed that there was an upregulation of SPX1a and SPX1b observed in the Area 2 of the brain of chronic social defeat stress fish compared to non-stress (control) fish (SPX1a, *p* = 0.023; SPX1b, *p* = 0.019) (Fig. 4A,B).

## Discussion

In contrast to the wide distribution of SPX1 mRNA in the brain of teleost^26,27^, our study showed highest expression of SPX1a mRNA in Area 2 (optic tectum, hypothalamus and midbrain) of the brain, which includes the midbrain where SPX1a ISH signals were localized. More specifically SPX1a mRNA expressing cells were seen in the ventromedial nucleus of TS in the midbrain.

In the goldfish and zebrafish, SPX immunoreactive cells have been reported in the medial longitudinal fasciculus of the brain^26,27^. These results contradict our localization study primarily because these studies use polyclonal antibody raised against human SPX1 while our study used the RNA probe specific to tilapia SPX1a for ISH. It is possible the human SPX antibody might recognise an epitope of a closely related peptide to SPX in these species. Alternatively, it is possible that in Nile tilapia, SPX1 has distinct localization compared to other teleost species. Interestingly, SPX1 mRNA expression has been reported in the dorsal habenula of the zebrafish^28^. We could detect low copy numbers of SPX1a using qPCR but failed to localize SPX1a mRNA expressing cells in the habenula. This suggest that the habenula of tilapia either lacks SPX1a or the expression levels depend on a physiological state of tilapia not tested in this study.

The TS in the midbrain of non-mammalian vertebrates is the homolog of the inferior colliculus (IC) in mammals^29^ which serves as a gateway to regulate sensory signal from the lower brain stem to the motor and endocrine regulatory centers in the forebrain^30^. In non-mammalian vertebrates the TS has been linked to social decision-making network involved in the control of defensive behavior through GABA, glutamate and the 5-HT systems^31–33^. Therefore, it can be speculated that SPX1a present in the TS might be involved in the control or regulated by these neurotransmitter systems.

SPX2, reported only in non-mammalian vertebrates^2,9^, was absent in the tilapia/cichlid genome^3^. The newly identified SPX1b is unique to cichlids and therefore limited information is available about its distribution and physiological function. ISH showed SPX1b mRNA expressing cells localized in the dorsal telencephalon, as well as the highest SPX1b mRNA levels was detected in Area 1(telencephalon and preoptic area) of the brain. The presence of SPX1b in the telencephalon suggest that it might be involved in feeding and avoidance behaviour since the telencephalon is part of the feeding and avoidance circuitry in teleost^34,35^.

In the present study, we do observe an upregulation of SPX1a and SPX1b in Area 2 during the chronic social defeat. The results from localization study revealed that SPX1b was localized in the telencephalon, whereas the upregulation of SPX1b expression level after chronic social defeat was observed in Area 2 (hypothalamus and midbrain). This could be due to the difference in sensitivity and detection limit of real-time PCR and ISH. The real-time PCR data revealed that SPX1b copy numbers in the brain is generally low, and perhaps below the detection range of ISH. Another speculation could be that the upregulation of SPX1b in Area 2 could be due to increased release of mRNA transcripts from the cell body in telencephalon (Area 1) and trafficked to the axonal terminals in Area 2 where it may be locally translated. Such mRNA transport and translation at axonal terminals has been shown for tyrosine hydroxylase and vasopressin^36,37^. Nevertheless, the fact that both SPX1a and SPX1b was upregulated in brain Area 2 (optic tectum, hypothalamus and midbrain) indicates that these brain regions are sensitive to stress challenge after repeated social defeat. As chronic stress has been associated with negative impact on reproduction, growth, and feeding activity^22,38,39^, the upregulation of SPX mRNA expression levels under chronic social defeat stress suggests that SPX could act as an inhibitor in these physiological functions. A recent study in the zebrafish showed SPX1 neurons are GABAegic in nature that inhibit cellular signaling via GALR2 in the spinal cord^9^. Similarly, in tilapia SPX1a/b, GALR (2a and 2b) could inhibit and deregulate the hypothalamic–pituitary–gonadal (HPG) axis.

The plasma cortisol level increased after chronic defeat stress in this study. In tilapia, a large population of CRH neurons have been reported in the telencephalon where SPX1b expressing cells are localized^40^. Similarly, low density of CRH fiber projections and glucocorticoid receptors have been reported in the TS area where SPX1a expressing cells are localized^40,41^. The upregulation of SPX1 mRNA expression levels in chronic social defeat stress fish (elevated plasma cortisol) suggest that SPX1 may be under the feedback regulation of cortisol. The increase in gene expression of SPX1a observed during chronic social defeat evoked by prolong high level of plasma cortisol may also be indirectly mediated by an unknown factor. One of the possible regulators of SPX1a might be 5-HT. Although we did not measure the changes in the 5-HT level in the current study, previous studies have shown elevated serotonergic activity in the brain following chronic social defeat stress^42,43^. For instance, chronic social defeat experience in the rats increases the expression of both tryptophan hydroxylase 2 and serotonin transporter expression in the dorsal raphe nucleus ^44,45^. Similar studies in the rainbow trout and Nile tilapia have demonstrated activation of 5-HT system, characterized by high 5-HIAA/5-HT turnover rate, following social defeat stress exposure^19,25,46^. This increase in 5-HT activity was observed in both dominant and defeated fish during dominance establishment. After the establishment of dominancy, the 5-HT activity returned to baseline in the dominant fish, while continued to be elevated in the defeated fish^47^.

The TS area in teleost is densely innervated by 5-HT fiber projections^48,49^. Indeed, our preliminary observations show that the area where SPX1a expressing neurons are localized is densely innervated by 5-HT fiber projections (unpublished data). Since neurons of the ventral periventricular pretectal nuclei (PPV) project to the TS in the brain of tilapia^50^, this suggests an indirect neuroanatomical link between the 5-HT neurons in the PPV and SPX1a neurons localized in the ventromedial nucleus of TS can be speculated.

In summary, we localized SPX1a and SPX1b -positive cells in the brain of Nile tilapia using ISH. SPX1a expressing cells were located in the TS of the midbrain region, while SPX1b expressing cells were localized in the telencephalon. In the present study, SPX1a and SPX1b mRNA levels were up-regulated in Area 2 region which include the optic tectum, hypothalamus and midbrain of the chronic socially defeated stress tilapia with elevated plasma cortisol level. This suggests that repeated social defeat stress probably through an intermediate 5-HT system regulates SPX1a and SPX1b expression.

## Material and methods

### Animals

Adult male Nile tilapia (n = 42) were kept in a fish aquarium (size: 90 × 45 × 45 cm) supplied with constant aeration and circulating freshwater. The fish was obtained from Intersea Fishery (Malaysia) Sdn Bhd. The environment of the aquaria was set to resemble their natural habitat (28 ± 0.5 °C; 14hL: 10hD cycle). The fish were fed cichlid pellet three times a day. All experimental procedures were approved and performed under the guidelines given by the Monash University Animal Ethics Committee (AEC) (MARP/2015/109; MUM/2018/02).

### Social defeat stress animals

Dominant fish were selected, which displayed aggressive social behaviors and had a typical pinkish body coloration. Only male tilapia (length: 15–16 cm) were used as dominant fish. The subject fish was selected from the community tank (male, length: 11–12 cm, n = 7). For chronic social defeat experiment, the dominant fish was moved into the experimental tank at 12 pm to establish dominance and to hold territory. After one hour (1 pm), the subject mature male fish was randomly chosen from the community tank to the experimental tank. Social interaction was then allowed, and the number of attacks was observed by an investigator. Once the number of attacks received by the subject fish reached 200, a partition which allow visual contact, was inserted into the experimental tank to separate the dominant fish from the subject fish. The experiment ended after 3 hours (3 pm) and the subject fish was moved to another tank and maintained there for rest of the day. The protocol was repeated for a total of five days, and the dominant fish were alternated to avoid exhaustion. The subject fish was anesthetized and decapitated for sample collection on the fifth day (3 pm). The social defeat paradigm is summarized in Fig. 1.

### Controls (non-stressed animals)

For the chronic social defeat experiment, the control was moved from the community tank to undergo the same protocol as the subject fish, but with the exception of the presence of a dominant fish. 9 controls and 7 socially defeated tilapia were included for gene expression analysis.

### Measurement of plasma cortisol levels

The plasma cortisol measurement was carried out using high performance liquid chromatography (HPLC). Sample preparation and subsequent protocol for HPLC measurement of plasma cortisol in tilapia blood are as described previously^25^. Briefly, the plasma samples were obtained from tilapia blood by centrifuging at 3000 rpm for 10 minutes. Then, cortisol was extracted from the blood plasma with dexamethasone (Nacalai Tesque, Kyoto, Japan) as the internal standard. The standard solution of cortisol was prepared by dissolving 10 mg of cortisol powder (Nacalai Tesque) in 10 mL of methanol (Fisher Scientific UK Ltd). Next, the standard solution of cortisol was diluted in 55% methanol with dexamethasone (500 ng/mL; internal standard) to produce a serial of standard solutions in the range of 100, 200, 300, 400 and 500 ng/mL to assess the assay linearity. Subsequent chromatographic separation was carried out in the HPLC-DAD system (1260 Infinity, Agilent Technologies, CA, United States). A ZORBAX Rapid Resolution Eclipse Plus C18 column (4.6 mm × 75 mm, 1.8 μm, Agilent Technologies) was used and the temperature was maintained at 30 °C at a flow rate of 0.8 mL/min. A mixture of methanol-water (55/45; v/v) was used as the mobile phase. The eluent was monitored by UV absorbance at 245 nm. The injection volume is 20 μl. ChemStation software (RRID: SCR_015742, Agilent Technologies) was used to for raw data acquisition and processing.

### Gene expression analysis

Before decapitation, male Nile tilapia were anaesthetized in water supplemented with 0.02% benzocaine solution (Sigma, St.Louis, MO, USA). The brains were quickly collected and dissected immediately into three areas (Area 1: telencephalon, preoptic area; Area 2: optic tectum, midbrain, hypothalamus; Area 3: cerebellum, and hindbrain), preserved on dry ice and stored at −80 °C. Brain tissues were homogenized using the TRIzol reagent, and total RNA was extracted. RNA isolation was performed using the chloroform/isopropanol and 75% ethanol. Finally, the RNA pellet was dissolved in 20 μL of RNAse-free water. The collected RNA sample (initial total concentration = 1000 ng) was reverse transcribed into cDNA by using High Capacity cDNA Reverse Transcription Kit (Applied Biosystems). The cDNA samples were stored at −20 °C. Quantitative real-time PCR reaction was carried out on 7500 Fast Real-Time PCR system (Applied Biosystems) by using the SensiFAST SYBR Hi-ROX Kit (Bioline, Taunton, MA, USA). The cycling condition were: holding stage at 95 °C for 2 min, followed by cycling stage at 95 °C for 5 s and 63 °C for 30 sec for 40 cycles. The plasmid containing the sequence of gene of interest was diluted at concentration of 10^9^, 10^8^, 10 ^7^, 10^6^, 10^5^, 10^4^, 10′ to generate a standard curve to determine the absolute copy number of mRNA. The primer sequences were as follows: tilapia SPX1a: 5′-AAGGGCTCATTCCAGCGAAG-3′ and reverse primer, 5′-CGAGTCTCTAGGTGAAGAGTGTC (PCR product size 131 bp, GenBank accession number MN399182); tilapia SPX1b: Forward primer, 5′-GGCCATCCTATACCTGAAAGGA-3′ and reverse primer: 5′-GTTCTGCTTGTGAGTCCCTGAGT-3′ (PCR product size 103 bp, GenBank accession number MN399183). The primer efficiency for SPX1a and SPX1b is 85% and 80% respectively. Elongation Factor 1-Alpha (EF1A) gene was selected as the housekeeping gene for chronic social defeat experiment (forward primer: 5′ -GGACTGGCTTATGCTGATT-3′; Reverse primer: 5′-ACTGAGAAGAGGCACTGT-3′; PCR product size 105 bp; GenBank accession number NM_001279647.1) All data were calculated by absolute quantification and normalized to housekeeping gene.

### RNA probe synthesis

A 301 bp fragment of the Nile tilapia SPX1a (GenBank accession number MN399182, corresponding to nucleotide 88–388) and 304 bp fragment of the Nile tilapia SPX1b was generated by PCR from the Nile tilapia whole brain cDNA and cloned into a pGEM T-Easy Vector (Promega, Madison, WI, USA). The primer sequences were as follows: SPX1a: forward primer, 5′-GGGTTTGAAGACCGTCACAATA-3′ and reverse primer, 5′-TTCATCAGCTCCCTCTCTAGC-3′; SPX1b: forward primer, 5′-AGGAGAGCACAACGCTTAAAGTAAG-3′ reverse primer, 5′-GATTGCCTGCATCTTCTTCTACAG-3′. The sequence of the probes was confirmed by sequencing (BigDye Terminator v3.1 Cycle Sequencing kit; Applied Biosystems, Foster City, CA, USA) and 3310 Genetic Analyzer (Applied Biosystems). The plasmid was linearized with Sal1 or BSP191 restriction endonuclease; and antisense and sense riboprobes were synthesized using MAXIscript T7 and Sp6 RNA polymerase *In Vitro* Transcription Kit (Ambion, Austin, TX, USA). Labeling of the linearised riboprobe uses digoxigenin (DIG)-RNA labeling mix (Roche Diagnostics, Basel, Switzerland).

### *In-situ* hybridization (ISH)

Nile tilapia (n = 6) were anaesthetized by immersion into 0.02% benzocaine solution (Sigma, St. Louis, MO, USA). The fish were then decapitated, and the brains were collected and quickly fixed in buffered 4% paraformaldehyde at 4 °C for 6 h. The brains were cryoprotected in 20% sucrose at 4 °C overnight, and then embedded in OCT compound (Leica, Wetzlar, Germany). A series of coronal sections (thickness = 15 μm) was performed on the cryostat (Leica CM 1860), and thaw-mounted onto silane-coated glass slides (Muto Pure Chemicals, Tokyo, Japan). Brain sections were subjected to permeabilization with 0.2 M HCL for 10 min followed by proteinase K (1 μg/mL) at 37 °C for 15 min and hybridized with DIG-labelled RNA probe (500 ng/mL) at 50 °C overnight in a humidified chamber. Next, the sections were washed with 2X saline sodium citrate (SSC) for 20 min at room temperature post-hybridization, followed by 2X SSC and 0.1X SSC (20 min each) at 55 °C and blocked with 2% normal sheep serum. The sections were incubated with alkaline phosphatase-conjugated anti-DIG antibody (1:500, Roche Diagnostics) and the signal of DIG-labelled RNA probes were developed with 4-nitroblue tetrazolium chloride/5-bromo-4-chloro-3-indolyl-phosphate (NBT/BCIP, Roche Diagnostics).

### Image analysis

After the detection procedure, the sections were mounted using Aquatex (Merck, Darmstadt, Germany). Each slide was captured with digital slide scanner (Zeiss MIRAX MIDI, Carl Zeiss, Oberkochen, Germany) equipped with bright field illumination using the Panoramic Scanner software (3DHISTECH, Budapest, Hungary) at 10x and 20x magnifications.

### Statistical analysis

All data are expressed as the mean ± SEM per group, and independent sample T-test was carried out using SPSS version 23. One-way ANOVA was used for multiple group comparison. Statistical significance was defined as *P* value less than 0.05

## Supplementary information

Supplementary information.
