# Supplementary material for: Chronic Social Defeat Stress Up-Regulates Spexin in the Brain of Nile Tilapia (Oreochromis niloticus)
Source: Sci Rep. 2020 May 6;10:7666. doi: 10.1038/s41598-020-64639-4 (PMC7203209; doi:10.1038/s41598-020-64639-4)
Supplement: Supplementary file 1 — Supplementary information. [file 41598_2020_64639_MOESM1_ESM.docx]

**Chronic Social Defeat Stress Up-Regulates Spexin in the Brain of Nile Tilapia (Oreochromis niloticus).**

**Authors: Chor Hong Lim^1^, Tomoko Soga^1*^, Berta Levavi-Sivan^2^ and Ishwar S. Parhar^1^**

1. Brain Research Institute,

Jeffrey Cheah School of Medicine and Health Sciences,

Monash University Malaysia, Bandar Sunway, 47500, Selangor, Malaysia.

2. Department of Animal Sciences, The Robert H Smith Faculty of Agriculture, Food, and Environment, Hebrew University of Jerusalem, Rehovot 76100, Israel

*Correspondence:

Tomoko Soga

tomoko.soga@monash.edu

**Keywords: stress, teleost, neuropeptide, torus semicircular.**

**Supplementary Data 1**

A)  **SPX1a**

Cloned

Predicted

Cloned

Predicted

Cloned

Predicted

Cloned

Predicted

Cloned

Predicted

Cloned

Predicted


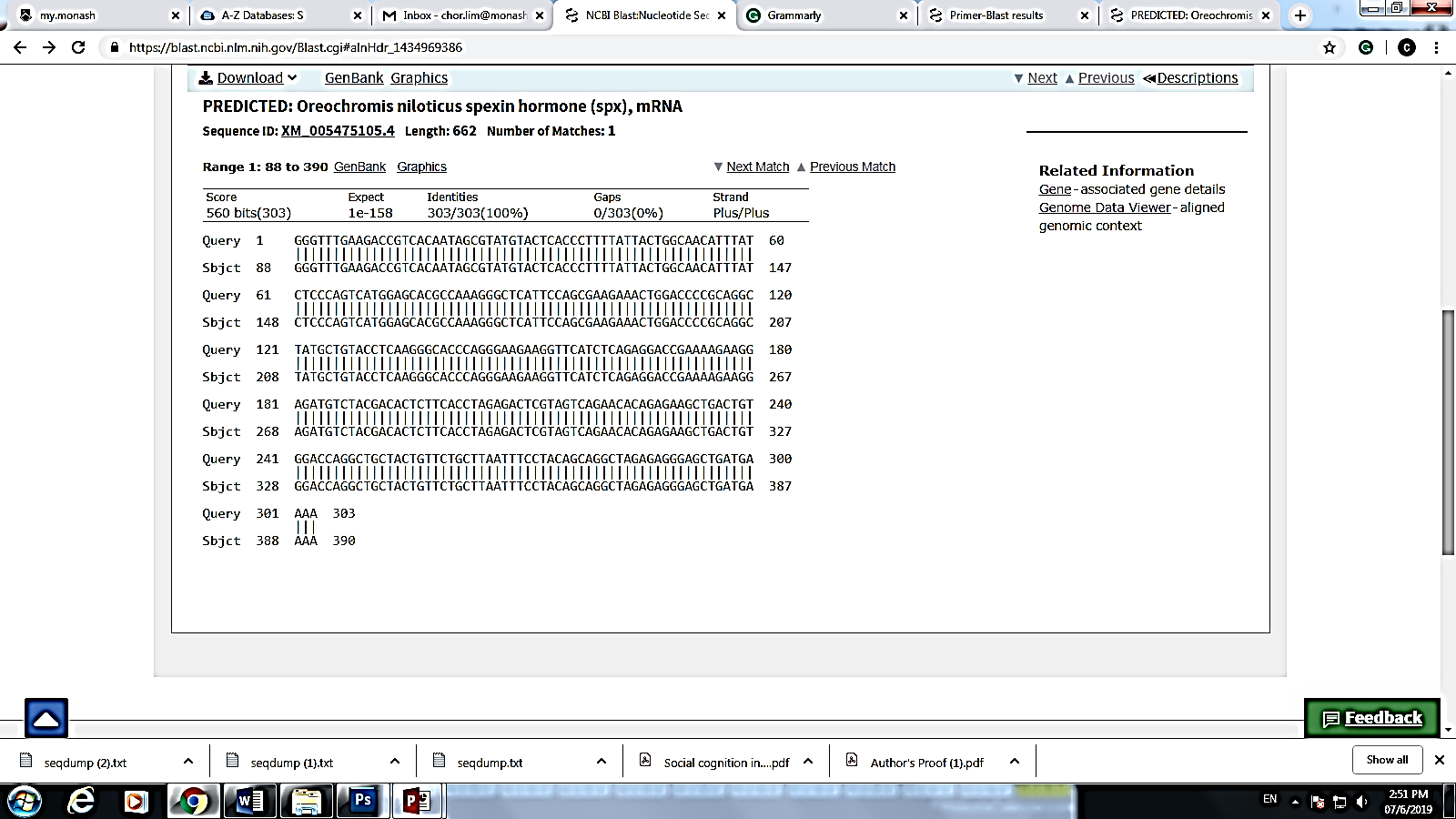


B) **SPX1b**

Cloned

Predicted

Cloned

Predicted

Cloned

Predicted

Cloned

Predicted

Cloned

Predicted

Cloned

Predicted


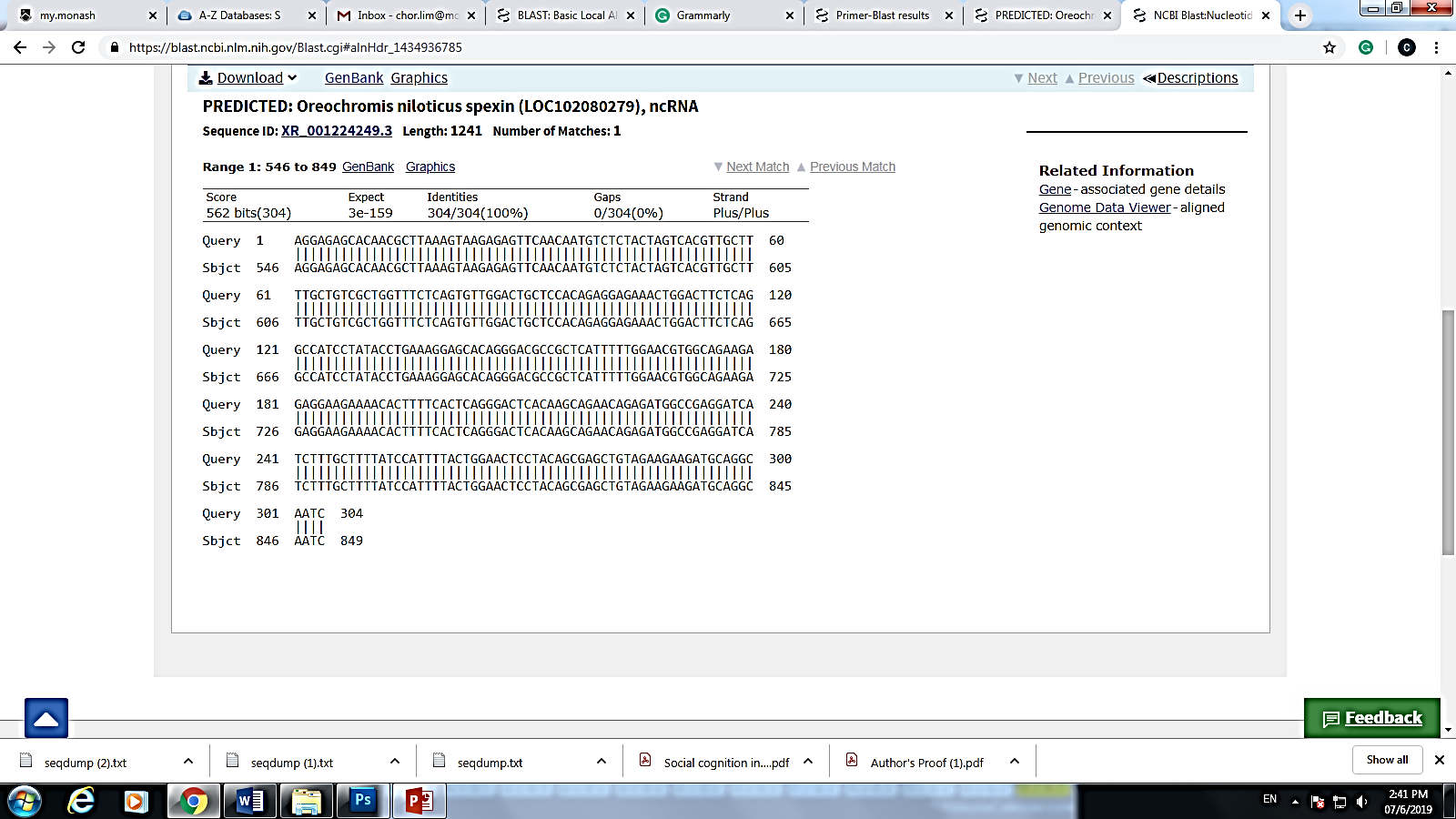
**Supplementary Data** **1** **Cloned sequence of SPX1a and SPX1b in Nile Tilapia A)** The amino acid sequence of the cDNA encoding SPX1a in Nile tilapia (GenBank accession number MN 399812). The cloned sequence showed 100% homology with the predicted sequence and correspond to position 88 to 390. **B)** The amino acid sequence of the cDNA encoding SPX1b (GenBank accession number MN 399813) in Nile tilapia. The cloned sequence is indicated in the uppercase, bold font and underlined section. The cloned sequence showed 100% homology with the predicted sequence and correspond to position 546 to 849.
